# 

**Published:** 1886-07

**Authors:** 


| | PAGE |
|---|---|
| ORIGINAL COMMUNICATIONS.—Febrile Intermittent Diarrhœa in Camels. Richard W. Burke. | 241 |
| How Can We Prevent False Hydrophobia? E. C. Spitzka, M. D. | 244 |
| The Effects of Lightning, Fire, Smoke and Steam upon Animals. Thomas Walley, M.R.C.V.S. | 267 |
| Relapsing Fever in Animals. John C. Peters, M. D. | 283 |
| EDITORIAL DEPARTMENT | 288 |
| PROCEEDINGS VETERINARY MEDICAL SOCIETIES | 293 |
| PROGRESS VETERINARY SCIENCE | 318 |
| REVIEWS | 325 |

